# Global analysis of the ovarian microRNA transcriptome: implication for miR-2 and miR-133 regulation of oocyte meiosis in the Chinese mitten crab, *Eriocheir sinensis* (Crustacea:Decapoda)

**DOI:** 10.1186/1471-2164-15-547

**Published:** 2014-07-01

**Authors:** Ya-Nan Song, Li-Li Shi, Zhi-Qiang Liu, Gao-Feng Qiu

**Affiliations:** Key Laboratory of Exploration and Utilization of Aquatic Genetic Resources Certificated by Ministry of Education, College of Fisheries and Life Science, Shanghai Ocean University, 999 Hucheng Huan Road, Pudong New Area, Shanghai 201306 China

**Keywords:** Ovary, MicroRNA transcriptome, Oocyte meiosis, Chinese mitten crab

## Abstract

**Background:**

MicroRNAs (miRNAs) are small non-coding RNA molecules that downregulate gene expression by base pairing to the 3′-untranslated region (UTR) of target messenger RNAs (mRNAs). Up to now, rare information for the miRNAs is available in decapod crustaceans. Our previous studies showed that many miRNA-binding sites are present in the 3′-UTR of the cyclin B in the Chinese mitten crab *Eriocheir sinensis*, suggesting that the translation or post-transcription of the crab cyclin B might be regulated by miRNAs during meiosis of oocyte.

**Results:**

To identify ovarian miRNAs in the mitten crab, ovarian small RNAs were subjected to high-throughput sequencing using an Illumina Genome Analyzer. Of 14,631,328 reads, 55 known miRNAs representing 44 miRNA families were identified and 136 novel miRNA candidates were predicted. The 5′ seed sequences of four miRNAs, miR-2, miR-7, miR-79 and miR-133, were revealed to complementary to miRNA binding sites in 3′-UTR of the cyclin B. Quantitative real time PCR analysis showed that miR-2 and miR-133 are much more abundant in the first metaphase (MI) of meiosis than in germinal vesicle (GV) stage. But their increasing expressions are independent of induction of gonadotropin-releasing hormone (GnRH). Further expression analysis using double-luciferase reporter genes assay showed that miR-2 and miR-133 can downregulate the 3′-UTRs of the crab cyclin B gene, indicating that they could inhibit the translation of the cyclin B. Western blot analysis confirmed that cyclin B protein is completely disappeared in fertilized egg at the metaphase-anaphase transition of meiosis I, suggesting that miR-2 and miR-133 could function in destruction of cyclin B near the end of MI.

**Conclusions:**

A high number of miRNAs have been identified from the crab ovarian small RNA transcriptom for the first time. miR-2 and miR-133 exhibit differential expression during the meiotic maturation of the oocytes and have activity in regulating the 3′-UTR of the crab cyclin B gene. This result is inconsistent with recent finding that miRNA activity is globally suppressed in mouse oocytes.

**Electronic supplementary material:**

The online version of this article (doi:10.1186/1471-2164-15-547) contains supplementary material, which is available to authorized users.

## Background

Small noncoding RNAs (snRNA) are distributed in a wide variety of species from plants to mammals. To date, several kinds of small RNAs, including microRNA (miRNA), small interfering RNA (siRNA), and Piwi-associated RNA (piRNA), have been discovered and demonstrated to function in various biological and cellular processes through regulation of gene expression at the post-transcriptional level [1–4]. The most characterized class of small regulatory RNAs is miRNAs, which are 19–23 nucleotides (nt) single strand RNAs and highly phylogenetically conserved across almost all species. Mature miRNAs are produced from longer miRNA precursors by two successive RNase III cleavages. After transcribed by RNA polymerase II, the primary miRNA transcripts (pri-miRNAs) are bound by the RNA-binding protein Dgcr8 (DiGeorge syndrome critical region 8) and initially processed by Drosha to form a ~70 nt hairpin-shaped pre-miRNA in the nucleus, and then the pre-miRNAs are transported to the cytoplasm where they are further processed by Dicer into an miRNA:miRNA* duplex. Only one strand of the duplex, the mature miRNA (miR), preferentially combined with the Argonaut protein to form a RNA-induced silencing complex (RISC). The miRNA then guides the RISC to their target mRNA through perfectly or imperfectly base-paring with the seed sequence, a 7–8 nucleotide section at the 5′ end of the miRNA. This binding either degrades the target RNA or blocks protein production, resulting in the suppression of the target gene expression [4–6]. Unlike miRNAs, piRNAs are 23–30 nt single-stranded RNAs, which are deprived from a Dicer-independent pathway and their sequences are poorly conserved among species [2]. piRNAs are abundantly expressed in the germ lines and interact with the PIWI subfamily of Argonaute proteins and repress the expression of selfish genetic elements, such as transposons [7].

A strict translational regulation is essential for oocyte maturation [8]. During oocyte maturation, oocytes that escape G2-arrest undergo germinal vesicle breakdown (GVBD). The expression of different maternal RNAs must be precisely spatio-temporally regulated in this process. Generally, the mRNA transcription is terminated in the GVBD-oocytes [9], since mature oocytes are arrested in the metaphase I (MI) or II (MII) of meiotic division until reactivation at fertilization. In Xenopus and mouse, large amounts of selective maternal mRNAs are deadenylated and translationally repressed during meiotic maturation [9–11]. The proteins being made during oocyte maturation are mostly cell cycle regulators needed to advance the oocyte through meiosis. Given that discovery of miRNAs and their roles in the regulation of gene expression through translational suppression or degradation of their target mRNA, there should be large numbers of such small RNAs in the ovary, which may be important regulators of gene expression during oocyte maturation.

Oocyte GVBD, meiotic maturation is governed by the maturation-promoting factor (MPF), cyclin B and cdc2 kinase complex, cyclin B synthesis and degrade during meiosis I, II. It has been demonstrated that gonadotropin-releasing hormone (GnRH) can induce oocyte maturation through regulating MPF activity [12, 13]. Cytoplasmic polyadenylation element (CPE) and translation control element (TCE) in the 3′-untranslated region (UTR) of cyclin B mRNA play important roles in the translational regulation of gene expression in clam, Drosophila, Xenopus and mouse [8, 14]. The CPE can activate translation of cyclin B during oocyte maturation by helping consort with the polyadenylation signal [15]. On the contrary, TCE in 3′-UTR is involved in translational repression of the transcripts in the pole cells of the early Drosophila embryo [16]. In our previous study, eight potential CPEs but no TCE are found in the unique long 3′-UTR of the cyclin B in the Chinese mitten crab *Eriocheir sinensis* [17]. Interestingly, the miRNA-binding sites, namely GY-box, Brd-box, and K-box motifs for translational regulation of the Enhancer of split Complex [E(spl)-C] and the Bearded-Complex [Brd-C] family genes in Drosophila [18], are simultaneously present in the 3′-UTR of the cyclin B in Chinese mitten crab [17], suggesting that the translation or post-transcription of the crab cyclin B might be regulated cooperatively via CPE and the miRNA-binding sites. To examine the crab ovarian miRNA expression and test whether miRNA can regulate the cyclin B expression, we generated an ovarian small RNA library and characterized the miRNA transcriptom by combining the Solexa sequencing with bioinformatics analysis. We identified a total of 191 miRNAs, including 55 known miRNAs and 136 novel miRNA candidates. miR-2 and miR-133 were revealed to have a role in the regulation of 3′-UTR of the crab cyclin B gene. This study represents the first survey of the crab miRNA in the ovary and provides the first evidence for the involvement of miRNA in the crab oocyte maturation.

## Results

### Global analysis of small RNAs in the crab ovary

**Figure 1:**
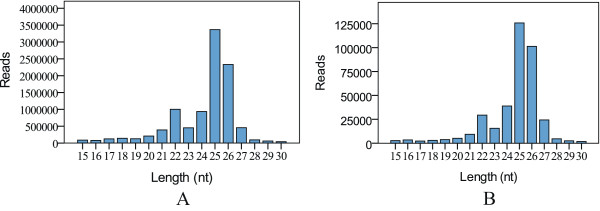
**Length distribution and abundance of small RNAs in the ovaries of the mitten crab.** Abundance of each size class of small RNAs based on nucleotide (nt) length plotted using total small RNA reads **(A)** and unique reads **(B)**.

To retrieve as many small RNAs as possible with less cost, a cDNA library of the crab small RNAs was constructed from a mixture of total RNAs from various ovarian stages and was high-throughput sequenced using the Solexa system. A total of 14,631, 328 reads were obtained from the sequencing machine. After discarding low quality reads, adaptor sequence, contaminating mRNA, rRNA, tRNA and snoRNA sequences by Illumina Pipeline filter (Solexa 0.3), 12,744,815 clean reads (87%) were obtained. Among the clean reads, 9,903,530 reads (73%) of sequences were found to be 15–30 nt in length and more than two copies, representing 374,055 unique sequences. As shown in Figure 1, the size (15–30 nt) of the small RNA from the crab ovary exhibited a distinct double peak distribution. One of peak for the 21 to 23 nt size class represents the typical miRNA of Dicer-processed product. Another peak for the 24 to 27 nt size class mostly represents longer piRNA-like small RNAs in the crab ovary (Figure 1). Obviously, the peak represented by the long size class (24–27 nt) was much higher than that for the shorter size class (21–23 nt), suggesting more abundant for the longer-sized small RNAs in the ovary.

### Identification of miRNAs in the crab ovaries

To identify potential miRNAs in the mitten crab ovary, the screened dataset were blasted against the known miRNA precursors (mirs) and mature miRNAs (miRs) of Arthropoda species in miRbase 16.0 (http://www.mirbase.org/) by miRAlign. Because the mitten crab genomic information is unavailable, a small crustacean Daphnia genome was selected as a reference one. Among the screened 9,903,530 reads, 395,080 reads that represent 2,359 unique sequences were mapped to the known mirs/miRs in miRBase 16.0 and/or Daphnia genome. The mappable sequences are divided into five groups: (1) 113,439 reads representing 466 unique sequences which were mapped to both Daphnia mirs/miRs in miRbase and Daphnia genome; (2) 29,863 reads representing 18 unique sequences which were mapped not only to the locations of known mir/miRs but also to other locations in the genome. The extended sequences at the mapped positions of the genome formed new hairpins; (3) 3,524 reads representing 30 unique sequences which mapped to both Arthropoda mirs and other genome, but unmapped to Daphnia genome; (4) 216,777 reads representing 425 unique sequences which were mapped to Arthropoda mirs, but unmapped to genome; (5) 31,477 reads representing 1420 unique sequences which were not mapped to Arthropoda mirs, but mapped to the genome. The extended sequences (60 nts on both directions) at the mapped genome positions have the propensity of forming hairpins. Finally, we identified 55 known miRNAs mapped to selected mirs/miRs (Additional file 1: Table S1) and 136 novel miRNAs un-mapped to selected mirs/miRs in miRbase (Additional file 2: Table S2). Among the known miRNAs, 53 belongs to 44 families, while the other two does not belonging to any family. These miRNAs in a family share the same seed sequence in the mitten crab as in other metazoan. There are some nucleotide substitutions in the flanking regions of the seed sequences in miR-275, miR-282, miR-305, miR-307 and miR-315. In addition, we identified seven miRNA* that aligned to 3′ end region of known miRNA precursors. The relative abundance of miRNAs varies greatly among the known miRNAs (Additional file 3: Figure S1). The top 4 most abundant miRNAs include miR-184, miR-100, miR-9b and let-7. Each of these miRNAs has more than 10,000 reads.

### Identification of the miRNAs targeting the crab cyclin B gene

**Figure 2:**
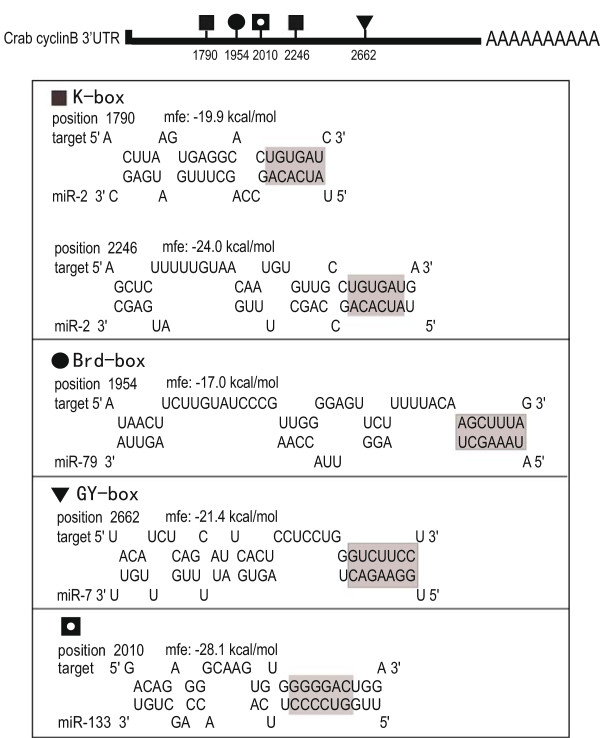
**The potential miRNA target sites of miR-2, miR-7, miR-79 and miR-133 in the 3′-UTR of the crab cyclin B as detected by RNAhybrid** [19]**.** Shadow indicated complementary region of seed sequence of the miRNA and their putative binding sites in the 3′-UTR. mfe: match free energy.

**Figure 3:**
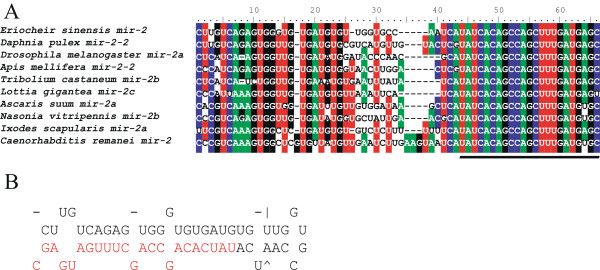
**The sequence and hairpin structure of the crab miR-2 precursor. (A)** Alignments of miR-2 precursor sequences from the mitten crab *E. sinensis* and other invertebrates including *Daphnia pulex*, *Drosophila melanogaster*, *Apis mellifera*, *Tribolium castaneum*, *Lottia gigantean*, *Ascaris suum*, *Nasonia vitripennis*, *Ixodes scapularis*, *Caenorhabditis remanei*. The identical nucleotides were shaded with color table. The mature miRNA sequence is underlined. **(B)** The hairpin structure of the crab miR-2 precursor predicted using the RNA-fold server on the website http://mfold.rna.albany.edu/?q=mfold. The mature miRNA sequence is shown in red.

To identify miRNAs targeting the crab cyclin B gene, the full sequences of 3′-UTR of cyclin B and miRNA candidates were submitted to RNAhybrid online searching (http://bibiserv.techfak.uni-bielefeld.de/rnahybrid/submission.html) based on the degree of miRNA:target sequence complementarity and the free energy level of RNA-RNA duplexes [19]. As shown in Figure 2, the 5′ seed sequences of miR-2, miR-7 miR-79 and miR-133 were revealed to be complementary to their corresponding target sites such as GY-box, Brd-box, and K-box in 3′-UTR of the crab cyclin B gene. The miR-133 was identified with the smallest free energy value (Figure 2). By taking advantage of a pair of primers designed according to the sequence of miR-2* and miR-2 (Additional file 4: Table S3), the precursor sequence for miR-2 was successfully amplified and cloned from the crab genomic DNA (Figure 3A) and its hairpin structure was predicted (Figure 3B). The crab miR-2 precursor shared high sequence homology with the miR-2 precursor from other invertebrates such as *Daphnia pulex* and *Drosophila melanogaster*.

### Quantification of the expression of the miRNAs in ovaries

**Figure 4:**
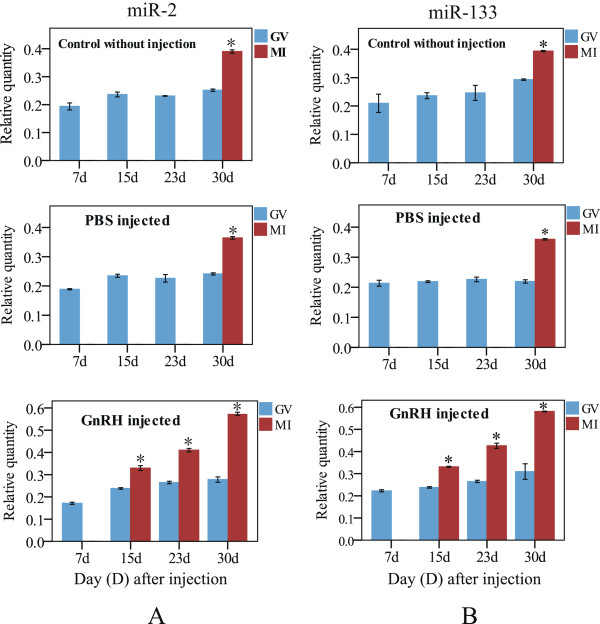
**Quantitative real-time PCR analysis of miR-2 (A) and miR-133 (B) expression in the ovaries of the mitten crab.** The crabs were injected with PBS solution or GnRH, and the blank control received no injection as shown on the top. The expression was normalized against U6 snRNA levels. Bar represents the standard error (SE) of the mean value for 3–4 individuals, Asterisks indicate significant difference (*P* < 0.05) between GV and MI stages.

To identify miRNAs differentially expressed during the crab oocyte maturation, the relative abundance of miR-2, miR-7, miR-79, miR-133 and other six selected miRNAs in the ovaries at GV and MI stages was assessed by quantitative real-time PCR. The selected miRNAs include top four most abundance miRNAs (miR-184, miR-100, miR-9b and let-7) in the ovaries and two miRNAs (miR-275 and miR-252) related to oogenesis in fruit fly [13]. Because our previous studies have shown that GnRH can induce GVBD of the crab oocyte (unpublished data), we further extended our investigation to the potential effect of the GnRH on the miRNA expression during the oocyte maturation. The expression level of the 10 miRNAs was measured over a period of 30 days after injection of GnRH. As indicated in Figure 4, GVBD was first observed at 15 days post injection of GnRH. No GVBD was found till 30 days in non-injected animals and control animals injected with PBS. After normalized against U6 snRNA, the relative expression level of miR-2 and miR-133 significantly increased at MI (Figure 4) whereas the other eight miRNAs exhibited stable expression from GV to MI among all the groups (data no shown).

To further test whether the differential expression of miR-2 and miR-133 at MI results from the induction of GnRH, we examined the detail expression profile of miR-2 and miR-133 in a period of 24-hour after injection of GnRH. Surprisingly, no significant difference of the expression level was found for the both miRNAs tested in 24 hours (data no shown), indicating GnRH cannot induce expression of miR-2 and miR-133. Therefore, we inferred that the increased expression of miR-2 and miR-133 observed at MI was independent of GnRH injection.

### Validation of regulation of the miRNAs for the 3′-UTR of the crab cyclin B gene

**Figure 5:**
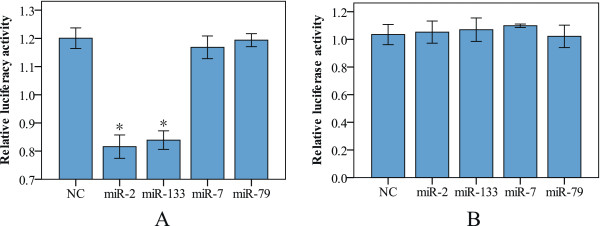
**Luciferase 3′-UTR reporter assays of the miRNAs silence effects using pGL3/cyclin B 3′-UTR (A) and pGL3/cyclin B 3′-UTR mutant (B) reporter vectors.** Firefly luciferase activity was evaluated 24 h after cotransfection. Values are mean ± SE of three independent experiments. Asterisks indicate significant difference (*P* < 0.05) compared with negative control (NC) using pGL3 empty vector.

To determine whether there are direct interactions between miR-2, miR-133, miR-7, miR-79 and their target sites of the crab cyclin B gene, we used a luciferase 3′-UTR reporter assay to measure the inhibitory effects of these miRNAs. The full sequence of the cyclin B 3′-UTR harboring GY-box, Brd-box, and K-box motifs or mutated boxes was cloned into the downstream of luciferase reporter gene. After cotransfected HEK 293 T cells, miR-2 and miR-133 mimics significantly reduce the luciferase activity from the reporter construct containing the cyclin B 3′-UTR, whereas miR-7, miR-79 and negative control mimics have no effect on the luciferase activity (Figure 5A). No effect was also detected with a construct containing mutated seed sites in the cyclin B 3′-UTR (Figure 5B). These results indicated that miR-2 and miR-133 can downregulate the target gene expression by miRNA binding sites in the 3′-UTR of cyclin B.

**Figure 6:**
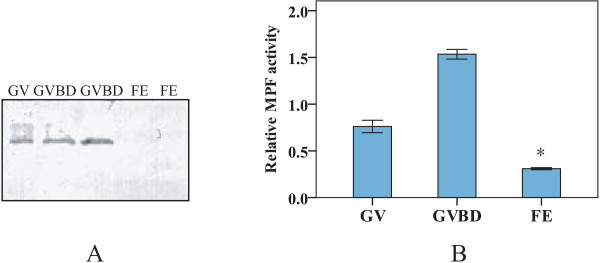
**Western blot analysis of the expression of the cyclin B protein (A) and MPF activity assay (B) in GV-, GVBD-oocytesand fertilized eggs (FE).** Asterisks indicate significant difference (*P* < 0.05) compared with those of GV-, GVBD-oocytes.

To verify whether the protein level of cyclin B drops during meiosis of oocytes, GV-, GVBD-oocytes and fertilized eggs were submitted to western blot analysis using an antibody against the crab cyclin B. The results showed that the crab cyclin B protein was present in GV- and GVBD-oocytes but disappeared in fertilized eggs at the time of transition from MI to anaphase of meiosis (Figure 6A), suggesting the potential role for the miR-2 and miR-133 in regulating the destruction of the cyclin B protein.

Furthermore, relative MPF activity was measured using a cdc2-cyclin B kinase assay kit. The data showed that MPF activity reach the highest level at the time of GVBD and sharply drop to the basal level at fertilization (Figure 6B), indicating that the destruction of the cyclin B protein in the fertilized egg results in a rapid and complete suppression of MPF activity.

## Discussion

Over thirty thousands of miRNA have now been identified in a multitude of organisms (miRBase version 20). However, there is rare information available regarding miRNA in decapod species. In this study, we attempted to identify small RNAs from the crab ovary as the first step toward understanding the regulatory roles of small RNAs in oogenesis. By combining the Solexa sequencing with bioinformatics analysis, we identified a total of 191 miRNAs, including 55 known miRNAs and 136 novel miRNA candidates. To the best of our knowledge, this is the first time that such a high number of miRNAs have been isolated from a decapod brachyuran. Furthermore, expression analysis revealed that miR-2 and miR-133 exhibited higher expression at MI arrest of meiosis and both of miR-2 and miR-133 can bind the 3′-UTR of the crab cyclin B, indicating that miR-2 and miR-133 is involved in the regulation of cyclin B expression during meiotic maturation of oocyte in the mitten crab.

### The survey of small RNAs in the crab ovary

A global survey of small RNAs in the crab ovary would contribute to understanding the function and evolution of small RNAs in crustacean. As in the insect species silkworm [20] and locust [21], our results indicate that there is a distinct bimodal distribution in the size of the small RNAs in the crab ovary at different developmental stages (Figure 1). One peak around 21–23 nt represents miRNAs and another distinct peak around 24–27 nt mostly represents longer piRNA-like small RNAs. The piRNA-like small RNAs make up a larger proportion in the crab ovary compared with miRNAs, indicating their potential functions in ovarian development, although we could not annotate them for the time being.

Given that the whole genome sequence data of the mitten crab are unavailable currently, it is essential for screening miRNAs to use reference genomes from other closely related species. Thus the Daphinia genome was employed as a reference in our analysis to identify the miRNAs from the crab deep sequencing data. The retrieved miRNAs shared conserved regions of the seed sequences, although there are some species-specific nucleotide substitutions in the flanking regions of the seed sequences in some miRNAs. Retrieval of miR-2*, together with predicted stem-loop structure for the precursor (Figure 3), strongly supported that the mature miRNA is processed from stem-loop structure. In the ovarian miRNA transcriptom, the reads of miRNAs sequences unevenly distributed within a wide range from 4 to 79,634 (Additional file 3: Figure S1). Quantitative real-time PCR assay showed that the expression of the top four most abundant miRNAs (miR-184, miR-100, miR-9b and let-7) is stable during the crab oocyte maturation. Intriguingly, previous studies in the fruit fly Drosophila showed that miR-184 plays a crucial role in the female germline development. Loss of miR-184 leads to multiple severe defects during oogenesis and early embryogenesis [12]. miR-100 and let-7 are involved in developmental timing in the nematode and the fly [22]. Further analysis of the expression profile of these miRNAs would ultimately lead to functional characterization of these abundant miRNAs in regulation of the crab ovarian development and early embryogenesis.

### miR-2 and miR-133 can regulate the 3′-UTR of the crab cyclin B gene

Accumulating evidence has showed that miRNAs are involved in regulation of cell cycle by controlling the expression of cyclins. For instance, miR-15b induced cell cycle arrest at G0/G1 phase by targeting cyclin E in glioma cells [23]; miR-16 induced G1 arrest partially by targeting cyclin D1 [24]; miR-122a can modulate cyclin G1 expression in human hepatocellular carcinoma-derived cell lines [25].

The regulative effect of miRNAs on mRNAs during meiotic maturation of oocytes was initially investigated in mice lacking Dicer. Dicer null oocytes showed abnormal gene expression and arrested with defects in spindle organization at MI stage, indicating that Dicer is essential for meiotic maturation of mouse oocytes [26, 27]. However, subsequent studies in Dgcr8 knockout mice revealed that limited miRNA-associated mRNA degradation and miRNA activity is globally suppressed in mouse oocytes and early embryos [28, 29], suggesting that the abundance of miRNAs in eggs may have no effect on the expression of their target genes. This data does not support the notion that miRNAs extensively modulate gene expression in mouse oocytes. The exact role of the miRNAs in the oocytes remains to be elucidated, since the fecundity of Dgcr8 knockout mice becomes diminished. Surprisingly, a recent study showed that miR-27a, which represses the translation of MAP2K4 and functions in the cell cycle, has activity in porcine oocytes, indicating that miRNA activity was not globally suppressed in porcine oocytes [30]. Furthermore, 15 human miRNAs were revealed to be differential expression in GV and MII oocytes [31], suggesting that miRNAs may play specific, rather than global, regulatory functions in gene expression during oocyte maturation.

As in many oviparous invertebrates, the mature GVBD-oocyte in the mitten crab arrests in MI till fertilization [32]. Fertilization is required for progression through meiosis I and completion of meiotic division. The present study showed that two miRNAs (miR-2 and miR-133) exhibited significantly increased expression from GV to MI stage (Figure 4). Interestingly, many putative binding sites for miRNAs including miR-2 and miR-133 were found in 3′-UTR of the crab cyclin B transcript (Figure 3). Thus we postulated that the high expression of miR-2 and miR-133 at MI arrest might be related to translation inhibition of the cyclin B. To test this hypothesis, the direct interactions between the miRNAs and their target sites of the crab cyclin B gene were examined using a luciferase 3′-UTR reporter assay. The result showed that miR-2 and miR-133 downregulated the luciferase activity by targeting the 3′-UTR of the cyclin B (Figure 5), indicating that miR-2 and miR-133 are involved in inhibiting the expression of the crab cyclin B gene. The result was confirmed by western blot analysis and MPF activity assay, in which the crab cyclin B protein disappeared and cyclinB-cdc2 kinase activity sharply dropped to the basal level in fertilized eggs at the transition from MI to anaphase I of meiosis, supporting the notion that cyclin B could be a direct target for miR-2 and miR-133 and the degradation of cyclin B is required for the fertilized egg to exit from MI to anaphase I of meiosis.

### GnRH can induce GVBD but has not effect on miR-2 and miR-133 expression

In vertebrates, reproductive success relies on the coordinated actions of the hypothalamic- pituitary-gonadal axis. GnRH is the central regulator of the reproductive hormonal cascade that is released from hypothalami into the hypophyseal portal system to stimulate the biosynthesis and secretion of gonadotropic hormones LH and FSH from pituitary. Several studies indicated that miRNA expression in reproductive tissues varied in response to pituitary and gonadal hormones [33]. Thirteen miRNAs were differentially expressed in periovulatory granulosa cells post-hCG (human chorionic gonadotropin) induction. Two miRNAs, miR-132 and miR-212 were found to be highly up-regulated following LH/hCG induction [33].

Our present studies showed that the GnRH injection in vivo can induce GVBD of the crab oocyte as in vertebrates. Although miR-2 and miR-133 displayed a higher expression at MI meiosis, no significant difference of the expression level was found for all the selected miRNAs including miR-2 and miR-133 in 24 hours post GnRH injection, indicating GnRH cannot induce expression of the miRNAs. There must be other unknown mechanism for regulation of differential expression of miR-2 and miR-133 during oocyte maturation. Also, more miRNAs need to be selected for assay in the future.

## Conclusions

A high number of miRNAs have been isolated from the crab ovary for the first time. This data provides basis for better understanding of miRNA roles in regulating the ovarian development. miR-2 and miR-133, which were predicted to target the crab cyclin B gene, displayed high expression in MI arrest stage relative to GV stage. Luciferase reporter genes assay demonstrated that miR-2 and miR-133 have activity and can downregulate the 3′-UTRs of the cyclin B gene. This result strongly suggest that miR-2 and miR-133 are involved in regulating the expression of the cycin B at the transition from MI to anaphase of meiosis I, and that miRNAs might have not been globally suppressed during meiosis of the crab oocyte.

## Methods

### Maintenance of crabs and collection of samples

Crabs were collected from a fisheries farm in Chongming Island (Shanghai, China), and were maintained in our laboratory in 150 L tanks equipped with air-lift circulating water. Crabs were daily fed clam tissue during the holding period. Ovarian samples were collected and immediately stored in liquid nitrogen and conserved at −86°C for isolation of total RNA. The ovarian tissues at different developmental stages were also fixed in Bouin’s fixative (75% picric acid, 25% formalin, 5% acetic acid) for histological observation. Ovarian developmental stages were classified according to histological characteristics and size of oocyte as described previously [17]. The different ovarian stages used in this study were previtellogenesis, vitellogenesis, late vitellogenesis (GV-stage), and final meiotic maturation (MI-stage). Fertilized eggs were collected from naturally spawning crabs. Crab assays were conducted in accordance with COPE (the Committee on Publication Ethics).

### GnRH injections

During breeding season, sexually mature females were selected for in vivo injections of synthetic GnRH (GL Biochem Ltd, Shanghai) in phosphate buffered saline (PBS) solution. Fifty individuals were injected at base of the fifth leg with 100 μL GnRH solution at the concentration of 500 ng GnRH/g body weight (BW). Another fifty crabs in a negative control group were injected with 100 μL PBS solution without GnRH. Experiment period lasted 30 days. Each individual was injected on a weekly basis. Ovarian samples were collected either at 6, 12, 24 hours post injection for short-term assay, or at 7, 15, 23, 30-day after injection for long term assay. Migration of germinal vesicle (GV) and GVBD were determined by placing oocytes in a clearing solution (formaldehyde, ethanol, acetic acid, 30:30:1) followed by microscopic examination.

### Small RNA library construction and sequencing

Total RNA was separately isolated from each stage using the Trizol reagent (Invitrogen) according to the manufacturer’s protocol. Concentrations of isolated RNA were quantified by measuring absorbance at 260 nm using a Nanodrop spectrophotometer (Thermo Scientific). Then equal quantities (10 mg) of total RNA isolated from different ovarian stages were pooled for library preparation. Small RNAs from 14 to 30 nucleotides were size-fractionated and purified from the total RNA pool with a Novex 15% TBE-Urea gel (Invitrogen). The purified small RNAs were ligated sequentially to 3′ and 5′ adaptors, after which ligation products were reverse transcribed using the primer on the 3′ adaptor and PCR amplified (15 cycles) using primers on both adaptors. Subsequently, PCR products were purified and small RNA library was sequenced on Illumina/Solexa G1 sequencer following the vendor’s recommended protocol.

### Bioinformatic analysis

Sequencing reads were processed using Illumina’s Genome Analyzer Pipeline software and the ACGT V3.1 program developed by LC Sciences (Houston, TX). After removing the sequences with low resolution, copy number less than three and contamination formed by the adaptor-adaptor ligation, filtering the low quality tags and the sequences mapping to the databases of mRNA (http://www.ncbi.nlm.nih.gov/), RFam (http://www.sanger.ac.uk/software/Rfam) and Repbase, the sequences were used to BLAST search against miRbase database (version16) to identify known miRNAs. The remaining sequences that did not match known miRNAs were mapped to Daphnia genome to identify potentially novel miRNAs. Novel miRNAs were predicted if the extended sequences at the mapped positions have the propensity of forming hairpin structures.

### Identification of miRNAs targeting the crab cyclin B gene

To identify the miRNAs targeting the crab cyclin B gene, the 3′-UTR of the crab cyclin B (Genbank number EU622123) and miRNA candidates were submitted to RNAhybrid online searching (http://bibiserv.techfak.uni-bielefeld.de/rnahybrid/submission.html). The prediction was performed with the default parameters such as the degree of miRNA:target sequence complementarity and the free energy level of RNA-RNA duplexes.

### Quantitative real-time PCR assay

MiRNAs expression levels were assayed by real-time PCR using a SYBR premix kit (TaKaRa) according to the manufacturer’s protocol. Total RNAs extracted from lVt (GV)- and MI-stage were reverse transcribed using M-MLV reverse transcriptase (Promega) and a stem-looped antisense primer. The resultant cDNA was submitted to the amplification of mature miRNAs using a miRNA specific primer and a universal primer. U6 snRNA gene was employed as an endogenous control. The primers for miRNAs and U6 snRNA are listed in Additional file 4: Table S3). Quantitative real-time PCR was conducted in 25 μl reaction volumes containing 300 nM of each primer and cDNA derived from 0.1 μg of total RNA. Cycling parameters were 95°C for 3 min, and followed by 35 cycles of 95°C for 35 s and 60°C for 30 s. All reactions were run in triplicate. The expression of miRNAs was normalized against U6 snRNA levels. The data are presented as means ± SE. Statistically significant differences were examined by paired *t*-test. A value of *P* < 0.05 was considered to be statistically significant.

### Luciferase 3′-UTR reporter assay

#### Vector construction

To construct the luciferase report vector, the crab cyclin B 3′-UTR (1611 bp) was PCR-amplified using sense primer 5′-CCG***CTCGAG***CCTCTTTCTCGTGAGTGTC-3′ and antisense primer 5′-CTA***GCTAGC***ATTCTTCTAACATTTGCGT-3′. The sense and antisense primers were supplemented with a *Xho I* (italics) and a *NheI* (italics) sites, respectively. After digested and purified, the amplified fragment was cloned downstream of firefly luciferase coding region at the *Xba I* site of pGL3 plasmid (Promega). Similarly, mutant cyclin B 3′-UTRs were generated by overlap extension PCR method and cloned into vector pGL3. All constructs were confirmed by sequencing.

#### Cell culture and transfections

Cell line HEK 293 T was obtained from the Chinese Academy of Science cell bank (Shanghai, China), and maintained in DMEM with 10% inactivated fetal bovine serum (Biowest), supplemented with glutamineand penicillin/streptomycin. Cells were seeded in a 24-well plate in antibiotic-free media one day prior to transfection. When the cells reached about 80% confluence, transfections were done using Lipofectamine 2000 reagent according to the manufacturer’s protocol (Invitrogen). The transfection mixtures contained 0.2 μg of pGL3 recombinant plasmid (or pGL3 negative control), 0.1 μg of pRL-TK-Control plasmid (Promega) and 0.03 μg of synthetic miRNA mimics (Bio Shanghai) in a final volume of 0.5 mL. Each sample was transfected in triplicate.

#### Luciferase activity assay

Cell lysates were harvested 24 hours after transfection, and luciferase activity was measured using the Dual-Luciferase Reporter Assay System (Promega). The knockdown levels of luciferase were normalized and shown by the ratio of relative luciferase activity. Statistical analysis of data was performed by one-way ANOVA using software Spss 10.0. *P* < 0.05 was considered significant. All *P* values were determined from two tailed tests.

### Western blot analysis

Western blot analysis was performed as previously described [32]. Briefly, GV-oocytes, GVBD-oocytes and fertilized eggs were homogenized in 3×SDS sample buffer (3% SDS, 1% β-glycerophosphate, 1 mM PMSF, 20% 0.05 M Tris–HCl, pH 8.0). The extractions were resolved on a 12% polyacrylamide gel and electroblotted onto a nitrocellulose membrane (Bio-Rad). The membrane was then blocked in 10% calf serum buffer and incubated with rabbit anti-cyclin B polyclonal antibody [34] at a dilution of 1:1000 for 1 h at room temperature. After being washed in TTBS buffer (1.21% Tris base, 0.9% NaCl and 0.11% Tween-80), the membrane was incubated with a second antibody (goat anti-rabbit serum) conjugated with horseradish peroxidase (Bio-Rad) at 1:10,000 for 2 h. Detection was performed using diaminobenzidine.

### MPF activity assay

MPF activity was measured using Cdc2-Cyclin B Kinase Assay Kit (Cat# CY-1164) according to the user manual provided by manufacturer (MBL).

## Electronic supplementary material

Additional file 1: Table S1: Novel miRNA candidates identified from the mitten crab ovary. (DOCX 16 KB)

Additional file 2: Table S2: Conserved miRNAs identified from the mitten crab ovary. (DOCX 19 KB)

Additional file 3: Figure S1: Number of high-throughput reads of the conserved miRNAs/miRNAs* in the crab ovary. (TIFF 3 MB)

Additional file 4: Table S3: The primers for miRNAs real-time RT-PCR. (DOC 4 MB)

## Abbreviations

MiRNA: MicroRNA
siRNA: Small interfering RNA
piRNA: Piwi-associatedRNA
UTRs: Untranslated regions
Dgcr8: DiGeorge syndrome critical region 8
RISC: RNA-induced silencing complex
nt: Nucleotides
EST: Expressed sequence tag
GnRH: Gonadotropin-releasing hormone
MPF: Maturation-promoting factor
CPE: Cytoplasmic polyadenylation element
TCE: Translation controlelement
PBS: Phosphate buffered saline
GV: Germinal vesicle
GVBD: Germinal vesicle breakdown.

## References

[1] Carrington JC, Ambros V (2003). Role of microRNAs in plant and animal development. Science.

[2] Lau NC, Seto AG, Kim J, Kuramochi-Miyagawa S, Nakano T, Bartel DP, Kingston RE (2006). Characterization of the piRNA complex from rat testes. Science.

[3] Watanabe T, Takeda A, Tsukiyama T, Mise K, Okuno T, Sasaki H, Minami N, Imai H (2006). Identification and characterization of two novel classes of small RNAs in the mouse germline: retrotransposon-derived siRNAs in oocytes and germline small RNAs in testes. Genes Dev.

[4] Ambros V (2004). The functions of animal microRNAs. Nature.

[5] Baek D, Villen J, Shin C, Camargo FD, Gygi SP, Bartel DP (2008). The impact of microRNAs on protein output. Nature.

[6] Yekta S, Shih IH, Bartel DP (2004). MicroRNA-directed cleavage of HOXB8 mRNA. Science.

[7] Khurana JS (2010). Theurkauf W: piRNAs, transposon silencing, and Drosophila germline development. J Cell Biol.

[8] Yamashita M (1998). Molecular mechanisms of meiotic maturation and arrest in fish and amphibian oocytes. Semin Cell Dev Biol.

[9] Paynton BV, Rempel R, Bachvarova R (1988). Changes in state of adenylation and time course of degradation of maternal mRNAs during oocyte maturation and early embryonic development in the mouse. Dev Biol.

[10] Su YQ, Sugiura K, Woo Y, Wigglesworth K, Kamdar S, Affourtit J, Eppig JJ (2007). Selective degradation of transcripts during meiotic maturation of mouse oocytes. Dev Biol.

[11] Varnum SM, Wormington WM (1990). Deadenylation of maternal mRNAs during Xenopus oocyte maturation does not require specific cis-sequences: a default mechanism for translational control. Genes Dev.

[12] Pati D, Lohka MJ, Habibi HR (2000). Time-related effect of GnRH on histone H1 kinase activity in the goldfish follicle-enclosed oocyte. Can J Physiol Pharmacol.

[13] Pati D, Habibi HR (2000). Direct action of GnRH variants on goldfish oocyte meiosis and follicular steroidogenesis. Mol Cell Endocrinol.

[14] de Moor CH, Richter JD (1999). Cytoplasmic polyadenylation elements mediate masking and unmasking of cyclin B1 mRNA. EMBO J.

[15] Huarte J, Stutz A, O'Connell ML, Gubler P, Belin D, Darrow AL, Strickland S, Vassalli JD (1992). Transient translational silencing by reversible mRNA deadenylation. Cell.

[16] Dalby B, Glover DM (1993). Discrete sequence elements control posterior pole accumulation and translational repression of maternal cyclin B RNA in Drosophila. EMBO J.

[17] Fang JJ, Qiu GF (2009). Molecular cloning of cyclin B transcript with an unusually long 3' untranslation region and its expression analysis during oogenesis in the Chinese mitten crab, Eriocheir sinensis. Mol Biol Rep.

[18] Lai EC, Tam B, Rubin GM (2005). Pervasive regulation of Drosophila Notch target genes by GY-box-, Brd-box-, and K-box-class microRNAs. Genes Dev.

[19] Kruger J, Rehmsmeier M (2006). RNAhybrid: microRNA target prediction easy, fast and flexible. Nucleic Acids Res.

[20] Jagadeeswaran G, Zheng Y, Sumathipala N, Jiang H, Arrese EL, Soulages JL, Zhang W, Sunkar R (2010). Deep sequencing of small RNA libraries reveals dynamic regulation of conserved and novel microRNAs and microRNA-stars during silkworm development. BMC Genomics.

[21] Wei Y, Chen S, Yang P, Ma Z, Kang L (2009). Characterization and comparative profiling of the small RNA transcriptomes in two phases of locust. Genome Biol.

[22] Rubio M, Belles X (2013). Subtle roles of microRNAs let-7, miR-100 and miR-125 on wing morphogenesis in hemimetabolan metamorphosis. J Insect Physiol.

[23] Xia H, Qi Y, Ng SS, Chen X, Chen S, Fang M, Li D, Zhao Y, Ge R, Li G, Chen Y, He ML, Kung HF, Lai L, Lin MC (2009). MicroRNA-15b regulates cell cycle progression by targeting cyclins in glioma cells. Biochem Biophys Res Commun.

[24] Liu Q, Fu H, Sun F, Zhang H, Tie Y, Zhu J, Xing R, Sun Z, Zheng X (2008). miR-16 family induces cell cycle arrest by regulating multiple cell cycle genes. Nucleic Acids Res.

[25] Gramantieri L, Ferracin M, Fornari F, Veronese A, Sabbioni S, Liu CG, Calin GA, Giovannini C, Ferrazzi E, Grazi GL, Croce CM, Bolondi L, Negrini M (2007). Cyclin G1 is a target of miR-122a, a microRNA frequently down-regulated in human hepatocellular carcinoma. Cancer Res.

[26] Murchison EP, Stein P, Xuan Z, Pan H, Zhang MQ, Schultz RM, Hannon GJ (2007). Critical roles for Dicer in the female germline. Genes Dev.

[27] Liu HC, Tang Y, He Z, Rosenwaks Z (2010). Dicer is a key player in oocyte maturation. J Assist Reprod Genet.

[28] Suh N, Baehner L, Moltzahn F, Melton C, Shenoy A, Chen J, Blelloch R (2010). MicroRNA function is globally suppressed in mouse oocytes and early embryos. Curr Biol.

[29] Ma J, Flemr M, Stein P, Berninger P, Malik R, Zavolan M, Svoboda P, Schultz RM (2010). MicroRNA activity is suppressed in mouse oocytes. Curr Biol.

[30] Chen L, Hu X, Dai Y, Li Q, Wang X, Xue K, Li Y, Liang J, Wang Y, Liu X, Li N (2012). MicroRNA-27a activity is not suppressed in porcine oocytes. Front Biosci (Elite Ed).

[31] Xu YW, Wang B, Ding CH, Li T, Gu F, Zhou C (2011). Differentially expressed micoRNAs in human oocytes. J Assist Reprod Genet.

[32] Qiu GF, Liu P (2009). On the role of Cdc2 kinase during meiotic maturation of oocyte in the Chinese mitten crab, Eriocheir sinensis. Comp Biochem Physiol B Biochem Mol Biol.

[33] Fiedler SD, Carletti MZ, Hong X, Christenson LK (2008). Hormonal regulation of MicroRNA expression in periovulatory mouse mural granulosa cells. Biol Reprod.

[34] Feng HY, Qiu GF (2011). Prokaryotic expression, antibody preparation, and identification of a cyclin B protein in the Chinese mitten crab Eriocheir sinensis and Malaysian giant prawn Macrobrachium rosenbergii. J Fish Sci China.

