# Supplementary material for: Global analysis of the ovarian microRNA transcriptome: implication for miR-2 and miR-133 regulation of oocyte meiosis in the Chinese mitten crab, Eriocheir sinensis (Crustacea:Decapoda)
Source: BMC Genomics. 2014 Jul 1;15(1):547. doi: 10.1186/1471-2164-15-547 (PMC4092226; doi:10.1186/1471-2164-15-547)
Supplement: Supplementary file 1 — Additional file 1: Table S1: Novel miRNA candidates identified from the mitten crab ovary. (DOCX 16 KB) [file 12864_2014_6223_MOESM1_ESM.docx]

Table S1 Conserved miRNAs identified from the mitten crab ovary

| ID | Sequence(5’-3’) | Homology | Length | Family |
| --- | --- | --- | --- | --- |
| ov1 | TAAGATCATTGTGAAAGCTGATT | bantam | 23 | bantam |
| ov2 | ACGTATACTGAATGTATCCTGA | miR-iab4 | 22 | miR-iab-4 |
| ov3 | TACGTATACTGAAGGTATACC | miR-iab8 | 21 | miR-iab-8 |
| ov4 | TGAGGTAGTAGGTTGTATAGTT | let-7 | 22 | Let-7 |
| ov5 | TGGAATGTAAAGAAGTATGGA | miR-1 | 21 | miR-1 |
| ov6 | TATCACAGCCAGCTTTGATGAGC | miR-2 | 23 | miR-2 |
| ov7 | TGGAAGACTAGTGATTTTGTTGTT | miR-7 | 24 | miR-7 |
| ov8 | TAATACTGTCAGGTAAAGATG | miR-8 | 21 | miR-8 |
| ov9 | TCTTTGGTTATCTAGCTGTATGA | miR-9a | 23 | miR-9 |
| ov10 | TCTTTGGTGATCTAGCTGTATGA | miR-9b | 23 | miR-9 |
| ov11 | TACCCTGTAGATCCGAATTTGT | miR-10 | 22 | miR-10 |
| ov12 | TGAGTATTACATCAGGTACTGGT | miR-12 | 23 | miR-12 |
| ov13 | TAGCACCATTTGAAATCAGTG | miR-29b | 21 | miR-29 |
| ov14 | GTGCATTGTAGTTGCATTGCA | miR-33 | 21 | miR-33 |
| ov15 | TGGCAGTGTGGTTAGCTGGTTGT | miR-34 | 23 | miR-34 |
| ov16 | TGAAAGACATGGGTAGTGAGATG | miR-71 | 23 | miR-71 |
| ov17 | ATAAAGCTAGGTTACCAAAGTTA | miR-79 | 23 | miR-9 |
| ov18 | GTGAGCAAAGTTTCAGGTGTGT | miR-87 | 22 | miR-87 |
| ov19 | TATTGCACTTGTCCCGGCCTGT | miR-92a | 22 | miR-25 |
| ov20 | AATTGCACTAGTCCCGGCCTG | miR-92b | 21 | miR-25 |
| ov21 | TATTGCACTCGTCCCGGCCT | miR-92c | 20 | miR-25 |
| ov22 | AACCCGTAGATCCGAACTTGTG | miR-100 | 22 | miR-99 |
| ov23 | TAAGGCACGCGGTGAATGCCAA | miR-124 | 22 | miR-124 |
| ov24 | TCCCTGAGACCCTAACTTGTGA | miR-125 | 22 | miR-125 |
| ov25 | TTGGTCCCCTTCAACCAGCTGT | miR-133 | 22 | miR-133 |
| ov26 | TTGCATAGTCACAAAAGTGATG | miR-153 | 22 | miR-153 |
| ov27 | TGGACGGAGAACTGATAAGG | miR-184 | 20 | miR-184 |
| ov28 | AGATATGTTTGATATTCTTGGTTG | miR-190 | 24 | miR-190 |
| ov29 | TACTGGCCTGCTAAGTCCCAA | miR-193 | 21 | miR-193 |
| ov30 | TGATTGTCCAAACGCAATTCTT | miR-219 | 22 | miR-219 |
| ov31 | CTAAGTACTAGTGCCGCAGGAG | miR-252a | 22 | miR-252 |
| ov32 | CTAAGTAGTAGTGCCGCAGGTA | miR-252b | 22 | miR-252 |
| ov33 | AATGGCACTGGAAGAATTCACGG | miR-263a | 23 | miR-263 |
| ov34 | CTTGGCACTGGAAGAATTCACAG | miR-263b | 23 | miR-263 |
| ov35 | TCAGGTACCTGATGTAGCGCGCGT | miR-275 | 24 | miR-275 |
| ov36 | TAGGAACTTCATACCGTGCTCT | miR-276 | 22 | miR-276 |
| ov37 | TGACTAGATCCACACTCATCCA | miR-279a | 22 | miR-279 |
| ov38 | TGACTAGATCTACACTCA | miR-279b | 18 | miR-279 |
| ov39 | TGACTAGATCCATACTCATCT | miR-279c | 21 | miR-279 |
| ov40 | TAGCCTCTCCTCGGCTTTGTCT | miR-282 | 22 | miR-282 |
| ov41 | TAGCACCATGTGAATTCAGT | miR-285 | 20 | miR-29 |
| ov42 | ATTGTACTTCATCAGGTGCTCGG | miR-305 | 23 | miR-305 |
| ov43 | TCAGGTACTGTGTGACTCT | miR-306 | 19 | miR-306 |
| ov44 | TCACAACCTCCTTGAGTGAGTGA | miR-307 | 23 | miR-67 |
| ov45 | TTTTGATTGTTGCTCAGAAGGC | miR-315 | 22 | miR-315 |
| ov46 | TGAACACAGCTGGTGGTATCTCAG | miR-317 | 24 | miR-317 |
| ov47 | CCAGATCTAACTCTTCCAGCTCA | miR-750 | 23 | miR-750 |
| ov48 | TAAGCGTATGGCTTTTCCCCT | miR-965 | 21 | miR-965 |
| ov49 | TTCGTTGTCGTCGAAACCTGCA | miR-981 | 22 | miR-981 |
| ov50 | GAAGCTCGTTTCTACAGGTATCT | miR-993 | 23 | miR-993 |
| ov51 | TGAGATTCAACTCCTCCAACTTAG | miR-1175 | 24 | miR-1175 |
| ov52 | TTGTGACCGTTATAATGGGCA | miR-2001 | 21 | miR-2001 |
| ov53 | TTGGTAACTCCACCACCGTTGGC | miR-2765 | 23 | miR-2765 |
| ov54 | GAGCTGCCCAATGAAGGG | miR-745 | 18 |  |
| ov55 | TATCCGGTTCGAAGGACCA | miR-2779 | 19 |  |
| ov56 | CTTGTCAGAGTGGGTGTGATGTG | miR-2* | 23 |  |
| ov57 | CATCTTACCGGACAGCATTAGA | miR-8* | 22 |  |
| ov58 | CAAATTCGGTTCTAGAGAGGTTT | miR-10* | 23 |  |
| ov59 | TCTCACTATCTTGTCTTTCA | miR-71* | 20 |  |
| ov60 | AAGAGAGCTATCCGTCGACAGT | miR-281* | 22 |  |
| ov61 | TTGAGATGGAGGACTCTTTGAAGGCC | miR-3228-as | 26 |  |
| ov62 | AGGACAGAACCCTGCGGAACAC | miR-3389* | 22 |  |
