# Supplementary material for: Global analysis of the ovarian microRNA transcriptome: implication for miR-2 and miR-133 regulation of oocyte meiosis in the Chinese mitten crab, Eriocheir sinensis (Crustacea:Decapoda)
Source: BMC Genomics. 2014 Jul 1;15(1):547. doi: 10.1186/1471-2164-15-547 (PMC4092226; doi:10.1186/1471-2164-15-547)
Supplement: Supplementary file 2 — Additional file 2: Table S2: Conserved miRNAs identified from the mitten crab ovary. (DOCX 19 KB) [file 12864_2014_6223_MOESM2_ESM.docx]

Table S2 Novel miRNA candidates identified from the mitten crab ovary

| ID | Sequence(5’-3’) | Length | Reads |
| --- | --- | --- | --- |
| 1 | CGCCACTCGTGCCGAGAGC | 19 | 249 |
| 2 | GCGGTGATGGGTGATGCC | 18 | 82 |
| 3 | CTCCCCTCTTCACCGG | 16 | 4 |
| 4 | AATGGACATCGACTTGCTAGAA | 22 | 4 |
| 5 | TTGGCTCGAAGATCTTGTAGAAGGC | 25 | 3 |
| 6 | CCGGAGGTAGGGTCCAGCGGCCG | 23 | 6 |
| 7 | GAGTGTCGCATCTGTGGGGAGTGTT | 25 | 3 |
| 8 | AATGTACCGTAGAATTTTGTGG | 22 | 3 |
| 9 | CAGGAATGGAACAAAAAGTGGCTGCT | 26 | 45 |
| 10 | TTTCGCGGTGGCGGCTCTAGCCC | 23 | 3 |
| 11 | AGGATGAACAGCTGGGTGAGCTGCA | 25 | 3 |
| 12 | CTCGGTCGGCGTCCTCC | 17 | 1348 |
| 13 | TTTCGTGGTGGCGGCTCTAGC | 21 | 34 |
| 14 | CTTCGCAGGGCAGAGACTCGCAGGG | 25 | 21 |
| 15 | TTTCGTATCTGGGCGGGACGTGGC | 24 | 4 |
| 16 | TGTGCTGTGCGCTGATTTAGGTCCCC | 26 | 8 |
| 17 | ATCTCACAGACGCGCCCGGTCC | 22 | 4 |
| 18 | CGGTCTGCCGTAGAGC | 16 | 63 |
| 19 | CCCCGACCGGCCGCTGCCGCC | 21 | 10 |
| 20 | TGAGTTGAAGAGTCGGTTCGGC | 22 | 9 |
| 21 | CCGCCCATCCGCTGTGTCTCG | 21 | 16 |
| 22 | AGCCCCGGCGCGGCGTCCTCC | 21 | 20 |
| 23 | TTTCACCGTATGCCCTTGTCTTCTCC | 26 | 22 |
| 24 | CCAGACTGCTCGGTACCGGTA | 21 | 5 |
| 25 | TGACGGACTATGCAGGTAATATATG | 25 | 3 |
| 26 | AACTCTGTAGGGACCTGAAACTTT | 24 | 3 |
| 27 | AGGCCAAGCTCCCCTC | 16 | 805 |
| 28 | TGTAAATAATGAAGAGCACTGG | 22 | 6 |
| 29 | GAGGTGGGGCCAACGGCC | 18 | 7 |
| 30 | CCCGTCGCTTACCTCC | 16 | 349 |
| 31 | AAGCGGGGTCGAGGGGG | 17 | 20 |
| 32 | CGGGGCTGCCTCCTACTCCGCCGCC | 25 | 4 |
| 33 | GAAGAACTATGAATGTAAGGAATGT | 25 | 15 |
| 34 | TCTGCGTCACGGATGGAAAGGG | 22 | 4 |
| 35 | TCGTGACGTGTAGCATCTAGCC | 22 | 3 |
| 36 | TGGGTGTACTCTGAATAGGCAGCGT | 25 | 3 |
| 37 | GCTCTTGGATGCCGGGC | 17 | 15 |
| 38 | TACTCTTTGTTGTCTGTTGCTC | 22 | 5 |
| 39 | CGGCGCGGCGGGGCAGCGC | 19 | 5 |
| 40 | TATTGCGACGCTAGAG | 16 | 6 |
| 41 | ATAGTAAGGTCTGAGGCTAAGC | 22 | 26 |
| 42 | CTTCGGGCCTCGGTCGGCGTCCTCCG | 26 | 7 |
| 43 | GCGGCGTCCCGAGTACC | 17 | 82 |
| 44 | TGTTGAGTTTATGGCTGAAAAC | 22 | 5 |
| 45 | TGTTATCGTCGTTGTCAGCAGAGG | 24 | 163 |
| 46 | CGAGTAGGAGGGTCGCAGGGGTGAGC | 26 | 32 |
| 47 | TGAACTGTTCAAAAGTGTTGGA | 22 | 4 |
| 48 | TGCAGCCTCCAGCCTTGAGTCG | 22 | 8 |
| 49 | GCCTGTTTGAAAAGCCTC | 18 | 8 |
| 50 | AGCGGGCCTGGGTTCG | 16 | 55 |
| 51 | AGGGCGACTGAACCGG | 16 | 171 |
| 52 | AAGGCGGCCGGCGGAC | 16 | 153 |
| 53 | CACTCGTCGAGCGAGCAACTCTCCCG | 26 | 14 |
| 54 | GAAGACACTGTGTTGGACTGTG | 22 | 3 |
| 55 | TGAGCGATGCCGAGTCTTAGCCCGC | 25 | 3 |
| 56 | ATTGTCACGCTCCGAAC | 17 | 3 |
| 57 | GTTGCTGAGGACTTTGGCA | 19 | 10 |
| 58 | ATTAGTCTTTTGTCTTCCAGTG | 22 | 5 |
| 59 | GAACATGATGCTGAGGGCTGTA | 22 | 3 |
| 60 | ACGTCGCCGGTGTCCGCC | 18 | 11 |
| 61 | CAGAGTTTGCAGAAACCCTGGA | 22 | 3 |
| 62 | CGAGGACGTGCGGGATTTGCG | 21 | 3 |
| 63 | CCGCTGGGCTTTCGGC | 16 | 6 |
| 64 | GATCACCGCGTCGTTTGGGC | 20 | 79 |
| 65 | TGAGACTATAATTTTGATACC | 21 | 24 |
| 66 | CGCAGTCTCTCACCGCCTA | 19 | 25 |
| 67 | GCGGTCGAAGGGGGAAAC | 18 | 9 |
| 68 | TATTTCTCCCATCACTGTATTATAT | 25 | 4 |
| 69 | TAGTAGGCTAGGCTAAAGGAAC | 22 | 8 |
| 70 | TGGTTAAGGAGTGGCTCTCCCGTCTC | 26 | 3 |
| 71 | AGCGGCCGTCGGTGCG | 16 | 3 |
| 72 | CCAACGGCCCGGTGTGCGGC | 20 | 47 |
| 73 | AAGTAGACTTGGCTAAAAGAAC | 22 | 7 |
| 74 | TGGTTGCTGGAGGAGGAGAAGCAGG | 25 | 6 |
| 75 | ACCGAACTGGTAGCGTACTGGG | 22 | 6 |
| 76 | CCTCGTATGGAGTATGCATCTC | 22 | 8 |
| 77 | CTTGGACCGTCGCAAGACG | 19 | 24 |
| 78 | CGGAGTGGCCTAGCACGCGTGC | 22 | 32 |
| 79 | GGCGGGCTTTGGGTGGGGGTT | 21 | 3 |
| 80 | GGAACGCGCTAGGCTGC | 17 | 8 |
| 81 | CATATCCTCCCATCTGGCCTGG | 22 | 3 |
| 82 | TCTCATCCGGGTTCGTCC | 18 | 44 |
| 83 | AAGAGTCAGGCTGATGCGTCAC | 22 | 4 |
| 84 | CGGCGCGGCGTCCTCTGGTC | 20 | 9 |
| 85 | TTCGACGCCGTCGTGCA | 17 | 27 |
| 86 | GCAGGTGTCCTAAGGCCA | 18 | 11 |
| 87 | CTTGGGATCGGACTGATGTCGA | 22 | 5 |
| 88 | ACTATTTGACGGACGACTGTAG | 22 | 3 |
| 89 | CCGCAGGGTTCGCGCAGCGCCGGTC | 25 | 34 |
| 90 | TTTGACAAGGCTTTCGTAGGAG | 22 | 6 |
| 91 | TCCAGGATAGAGACCCCTCTGT | 22 | 28 |
| 92 | AGGACCGCAACTCTCTCA | 18 | 3 |
| 93 | GAGAAGTTTCGGGTCCCTACAGA | 23 | 14 |
| 94 | CACCTATAGACTGCTGAGTTGCTC | 24 | 13 |
| 95 | TGGGAGTTAGCCTGTGAAGC | 20 | 6 |
| 96 | TCTAACGGGCTGCTGGTGAGGCCTC | 25 | 5 |
| 97 | AGCGACGTTGGGTCTGGATAGTAC | 24 | 511 |
| 98 | TACGAAAGCCTTGTCAAATGTG | 22 | 3 |
| 99 | TCCGATTACAGCACCCAACTCTGGA | 25 | 108 |
| 100 | CCAGATGCTGTTGGCG | 16 | 92 |
| 101 | GCATTGCGATGGTCAGAGAT | 20 | 175 |
| 102 | TGCCTTCCGATCCTGCTCTGTC | 22 | 66 |
| 103 | CATACACATACACATACACA | 20 | 3 |
| 104 | AAATATCTATCCGTTTCCACTT | 22 | 8 |
| 105 | AGCCCAGCGCATAACCTGGCGTCG | 24 | 3 |
| 106 | ATTCATCGTCTCTGTACCCTTC | 22 | 3 |
| 107 | CCGGCGAGCAGCGACGGAGGCACGG | 25 | 5 |
| 108 | TCTTCCTCGTCTGATTGCTGCGTCT | 25 | 3 |
| 109 | CTGGATATCCCTTCACTGGTAG | 22 | 6 |
| 110 | TGTGTTCTCCCCTCAGCGTCACTGT | 25 | 4 |
| 111 | ATTTATGATGCGGGACTTTATC | 22 | 15 |
| 112 | CCCCTCGTGATGTAGGTCGCC | 21 | 89 |
| 113 | ACTGGACTTGACTGAAATGAAG | 22 | 4 |
| 114 | TAGAATGTTGGGCCGATCTCCA | 22 | 33 |
| 115 | GAAGGTCGTGGACTACCAGA | 20 | 3 |
| 116 | TGACTTCTTCATCACCGGCGGCGACC | 26 | 5 |
| 117 | TGTAGCACCGGTAGGC | 16 | 3 |
| 118 | GAACGCAAACTCCACCAAGGTT | 22 | 3 |
| 119 | TGGAGACACAGTATGATGACTC | 22 | 5 |
| 120 | CATCCCTGTTTCTACATTCTCT | 22 | 3 |
| 121 | TGTGTGCTGGTACTCATGAGTCTCCT | 26 | 14 |
| 122 | TGGACACGTCAGAAAACCACGG | 22 | 21 |
| 123 | CGTCCTCCGGTCGCTCCC | 18 | 22 |
| 124 | CCACGTGGGATTGCCTCCT | 19 | 234 |
| 125 | CCGAAGTGATAGCGTACTGGAC | 22 | 13 |
| 126 | CAACAATTGGATCTGTAGCTGC | 22 | 138 |
| 127 | ATTTTTTCTCATTTTTCTCGCT | 22 | 5 |
| 128 | GGCGGGCTTCGGGTGGGGGTT | 21 | 385 |
| 129 | CCAGGGGTGTTGCGTTTCGG | 20 | 5 |
| 130 | GATTGTCGTACTCAGCCTC | 19 | 4 |
| 131 | ATTAGTTAGACCTCATCTTGAC | 22 | 9 |
| 132 | CAGGATAGCTGGTACTCGC | 19 | 219 |
| 133 | TGTGTCTTGCTGTCGTAACCGCC | 23 | 4 |
| 134 | TTTACCATGTAGCAGCGAAGGG | 22 | 3 |
| 135 | TGTAAAGAACTATGAATGTAAGGAAT | 26 | 3 |
| 136 | TCTCTACTTCAAGTCTCTGGGCCTG | 25 | 4 |
