# Supplementary figures and images for: Global analysis of the ovarian microRNA transcriptome: implication for miR-2 and miR-133 regulation of oocyte meiosis in the Chinese mitten crab, Eriocheir sinensis (Crustacea:Decapoda)
Source: BMC Genomics. 2014 Jul 1;15(1):547. doi: 10.1186/1471-2164-15-547 (PMC4092226; doi:10.1186/1471-2164-15-547)

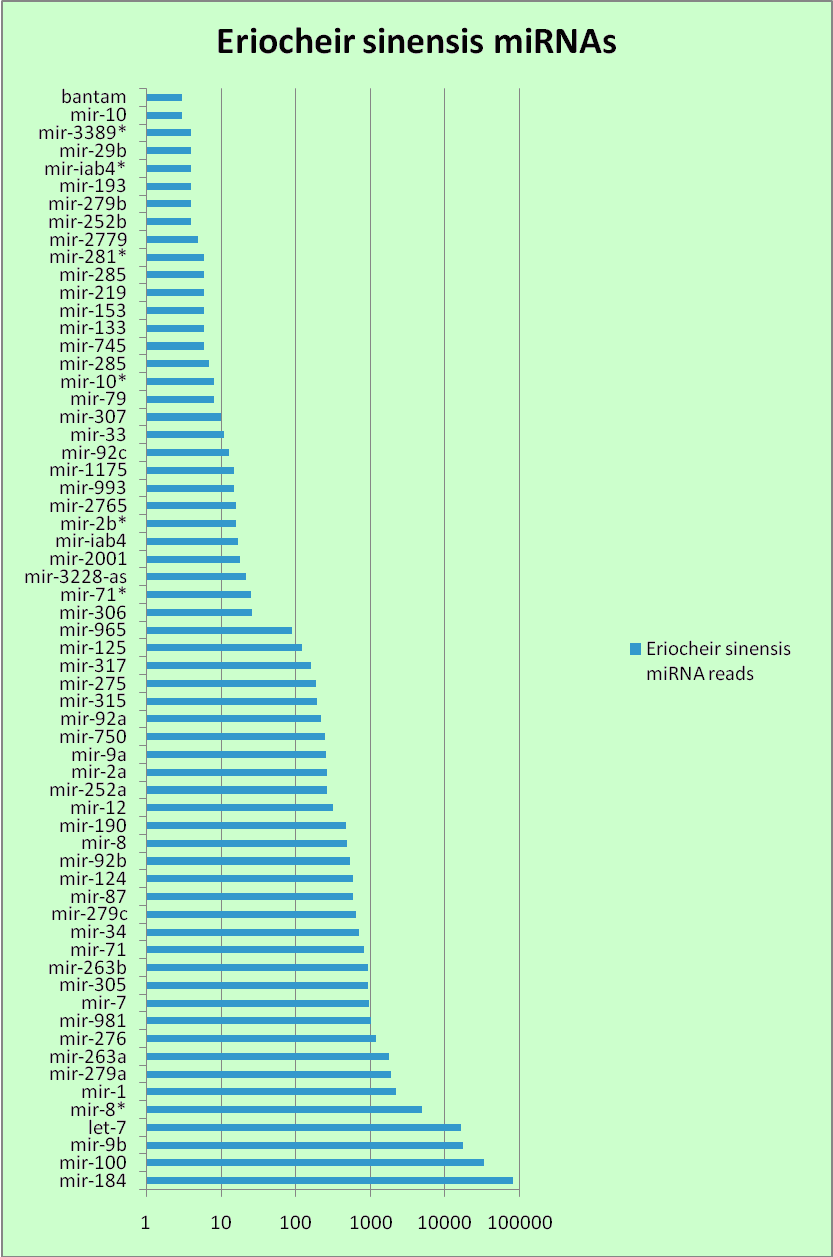

Supplement: Supplementary file 3 — Additional file 3: Figure S1: Number of high-throughput reads of the conserved miRNAs/miRNAs* in the crab ovary. (TIFF 3 MB) [file 12864_2014_6223_MOESM3_ESM.tiff]
