# Supplementary material for: Global analysis of the ovarian microRNA transcriptome: implication for miR-2 and miR-133 regulation of oocyte meiosis in the Chinese mitten crab, Eriocheir sinensis (Crustacea:Decapoda)
Source: BMC Genomics. 2014 Jul 1;15(1):547. doi: 10.1186/1471-2164-15-547 (PMC4092226; doi:10.1186/1471-2164-15-547)
Supplement: Supplementary file 4 — Additional file 4: Table S3: The primers for miRNAs real-time RT-PCR. (DOC 4 MB) [file 12864_2014_6223_MOESM4_ESM.doc]

## Table S3 The primers for miRNAs real-time RT-PCR

| Primer names | Primer sequences（5'-3'） |
| --- | --- |
| miR-184-RT | CTCAACTGGTGTCGTGGAGTCGGCAATTCAGTTGAGCCTTATCA |
| miR-184-F | ACACTCCAGCTGGGTGGACGGAGAAC |
| Let-7-RT | CTCAACTGGTGTCGTGGAGTCGGCAATTCAGTTGAGAACTATA |
| Let-7-F | ACACTCCAGCTGGGTGAGGTAGTAGGTT |
| miR-100-RT | CTCAACTGGTGTCGTGGAGTCGGCAATTCAGTTGAGGACAAGTT |
| miR-100-F | ACACTCCAGCTGGGAACCCGTAGATCCG |
| miR-9b-RT | CTCAACTGGTGTCGTGGAGTCGGCAATTCAGTTGAGTCATACAG |
| miR-9b-F | ACACTCCAGCTGGGTCTTTGGTGATCTAG |
| miR-2-RT | GTCGTATCCAGTGCAGGGTCCGAGGTATTCGCACTGGATACGACGCTCATCA |
| miR-2-F | CGGCGGTATCACAGCCAGCT |
| miR-7-RT | CTCAACTGGTGTCGTGGAGTCGGCAATTCAGTTGAGAACAACAA |
| miR-7-F | ACACTCCAGCTGGGTGGAAGACTAGTGAT |
| miR-79-RT | CTCAACTGGTGTCGTGGAGTCGGCAATTCAGTTGAGTAACTTTG |
| miR-79-F | ACACTCCAGCTGGGATAAAGCTAGGTT |
| miR-133-RT | CTCAACTGGTGTCGTGGAGTCGGCAATTCAGTTGAGACAGCTGG |
| miR-133-F | ACACTCCAGCTGGGTTGGTCCCCTTCAA |
| miR-252-RT | CTCAACTGGTGTCGTGGAGTCGGCAATTCAGTTGAGCTCCTGCG |
| miR-252-F | ACACTCCAGCTGGGCTAAGTACTAGTGC |
| miR-275-RT | CTCAACTGGTGTCGTGGAGTCGGCAATTCAGTTGAGACGCGCGC |
| miR-275-F | ACACTCCAGCTGGGTCAGGTACCTGATGTA |
| miRNAs-R (universal) | AACTGGTGTCGTGGAG |
| U6-RT | AACGCTTCACGATTTTGCGT |
| U6-F | CTTGCTTCGGCAGAACATATACT |
| U6-R | AACGCTTCACGATTTTGCGT |


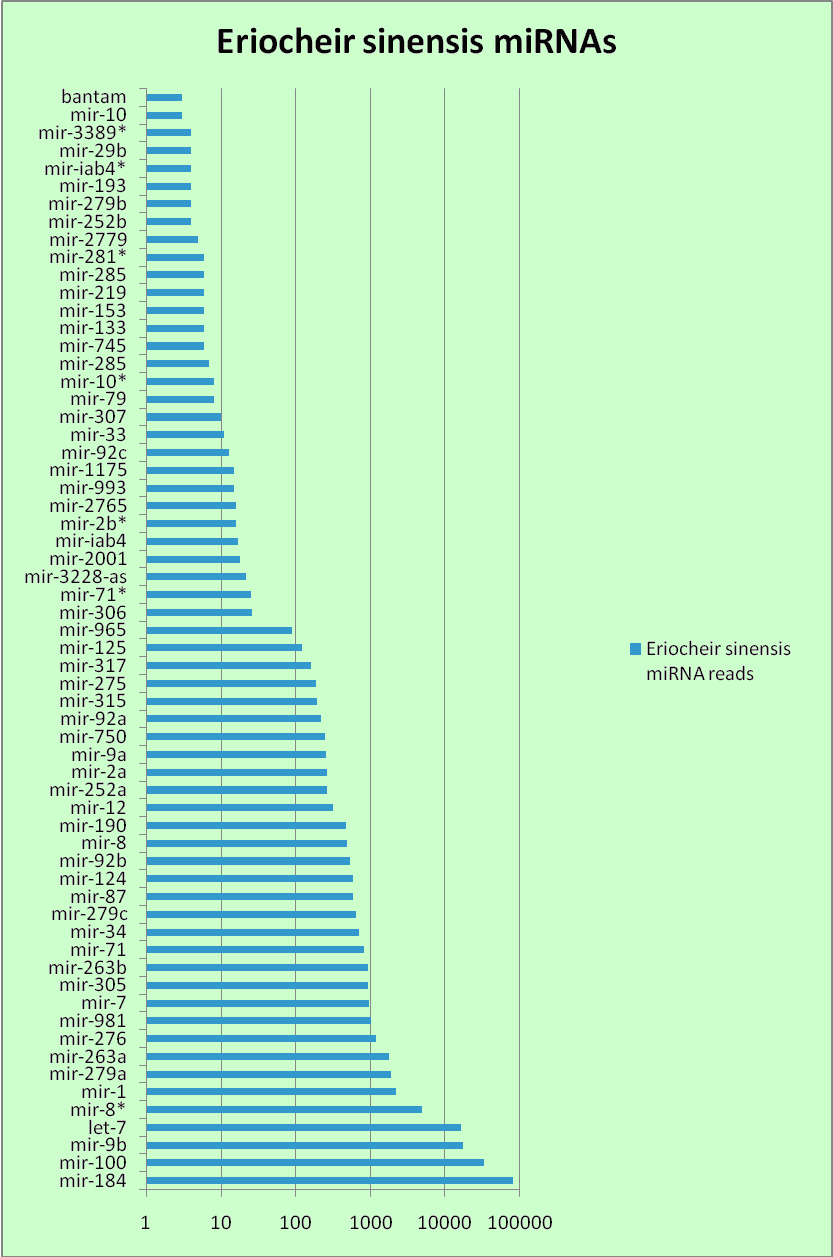


Fig.S1

Fig.1

Fig.2

Fig3

Fig.4

Fig.5

Fig.6
